# Type-4 Phosphodiesterase (PDE4) Blockade Reduces NETosis in Cystic Fibrosis

**DOI:** 10.3389/fphar.2021.702677

**Published:** 2021-09-08

**Authors:** Licia Totani, Concetta Amore, Antonio Piccoli, Giuseppe Dell’Elba, Angelo Di Santo, Roberto Plebani, Romina Pecce, Nicola Martelli, Alice Rossi, Serena Ranucci, Ida De Fino, Paolo Moretti, Alessandra Bragonzi, Mario Romano, Virgilio Evangelista

**Affiliations:** ^1^ Laboratory of Vascular Biology and Pharmacology, Fondazione Mario Negri Sud, Santa Maria Imbaro (CH), Mozzagrogna, Italy; ^2^ Laboratory of Molecular Medicine, Centre for Advanced Studies and Technology (CAST), Department of Medical Oral and Biotechnological Sciences, G. D’Annunzio University of Chieti-Pescara, Chieti, Italy; ^3^ Infection and Cystic Fibrosis Unit, Division of Immunology Transplantation and Infectious Diseases, IRCCS San Raffaele Scientific Institute, Milan, Italy; ^4^ Cystic Fibrosis Centre, S. Liberatore Hospital, Atri, Italy

**Keywords:** cystic fibrosis, neutrophil, neutrophil extracellular traps, lung damage, PDE4 inhibition

## Abstract

Neutrophilic inflammation is a key determinant of cystic fibrosis (CF) lung disease. Neutrophil-derived free DNA, released in the form of extracellular traps (NETs), significantly correlates with impaired lung function in patients with CF, underlying their pathogenetic role in CF lung disease. Thus, specific approaches to control NETosis of neutrophils migrated into the lungs may be clinically relevant in CF. We investigated the efficacy of phosphodiesterase (PDE) type-4 inhibitors, *in vitro*, on NET release by neutrophils from healthy volunteers and individuals with CF, and *in vivo*, on NET accumulation and lung inflammation in mice infected with *Pseudomonas aeruginosa*. PDE4 blockade curbed endotoxin-induced NET production and preserved cellular integrity and apoptosis in neutrophils, from healthy subjects and patients with CF, challenged with endotoxin, *in vitro*. The pharmacological effects of PDE4 inhibitors were significantly more evident on CF neutrophils. In a mouse model of *Pseudomonas aeruginosa* chronic infection, aerosol treatment with roflumilast, a selective PDE4 inhibitor, gave a significant reduction in free DNA in the BALF. This was accompanied by reduced citrullination of histone H3 in neutrophils migrated into the airways. Roflumilast-treated mice showed a significant improvement in weight recovery. Our study provides the first evidence that PDE4 blockade controls NETosis *in vitro* and *in vivo*, in CF-relevant models. Since selective PDE4 inhibitors have been recently approved for the treatment of COPD and psoriasis, our present results encourage clinical trials to test the efficacy of this class of drugs in CF.

## Introduction

Neutrophilic inflammation is the trademark of cystic fibrosis (CF) lung disease (Tirouvanziam, 2006). Excessive and persistent accumulation of neutrophils in the airways, associated with impaired bacterial clearance and tissue damage, is the early event in the life of patients with CF (Davis and Ferkol, 2013; Sly et al., 2013). In CF airways, neutrophils release proteases, mainly elastase that induces inflammatory responses, impairs ciliary function in epithelial cells, disables CXCL8-induced bacterial killing, and causes bronchomalacia and bronchiectasis (Hartl et al., 2007; Davis and Ferkol, 2013).

In addition, recruited neutrophils produce the so-called neutrophil extracellular traps (NETs), consisting of a nuclear DNA backbone decorated with granular enzymes that help to capture and kill, extracellularly, invading bacteria (Brinkmann et al., 2004). However, recent observations in preclinical and clinical CF models indicate that the excessive accumulation of NETs in the airways plays a key pathogenetic role in lung disease (Cheng and Palaniyar, 2013). Abundant NETs can be found in the airways of people with CF and significantly correlate with impaired lung function, suggesting that excessive NETosis, in CF, may act as a double-edged sword between host-defense and auto-inflammation (Marcos et al., 2015). Indeed, more recent observations show that *Pseudomonas aeruginosa* isolated from patients with CF is resistant to the bactericidal activity of NETs (Young et al., 2011). Moreover, excessive NET formation may play a pathogenetic role in vasculitis (Kessenbrock et al., 2009) and provide a scaffold for platelet adhesion and thrombus formation (Fuchs et al., 2010), thus mediating micro- and macrovascular occlusion.

The basic mechanisms of vital NETosis have been recently uncovered. Upon appropriate stimulation of neutrophils, the nuclear envelope disintegrates and allows mixing of chromatin with granular enzymes, such as myeloperoxidase and elastase, which together with type IV peptidyl-arginine deiminase (PAD) promotes chromatin decondensation before extracellular release of a large filament of DNA–enzyme complexes, as NETs (Neeli et al., 2008; Wang et al., 2009; Papayannopoulos et al., 2010; Thiam et al., 2020). Although the discovery of the process of NETosis and of the underlying mechanisms is relatively recent, its pharmacological modulation remains largely unknown. While PAD4 inhibitors, such as GSK484 (Mondal and Thompson, 2019), BMS-P5 (Li et al., 2020), or the more historic Cl-amidine, are described in the literature, they remain in a nonclinical stage so far. Thus, current therapies for CF lack specific approaches to tackle excessive NETosis.

Type 4 phosphodiesterases (PDE4s), the major isoform of PDEs expressed by myeloid cells, control a variety of inflammatory mechanisms in immune cells. In neutrophils, PDE4s are key mediators of cAMP degradation and, as a downstream effect, of neutrophil adhesion and migration, cytokine and chemokine release, synthesis of lipid mediators, and of reactive oxygen species (Sanz et al., 2005). In several animal models, genetic deficiency of PDE4 reduces neutrophilic inflammation (Jin and Conti, 2002; Ariga et al., 2004; Jin et al., 2005)*.* In agreement with genetic ablation, pharmacological blockade of PDE4 reduces leukocyte recruitment at the site of inflammation (Sanz et al., 2002; Sanz et al., 2007)*.* Moreover, it was reported that PDE4 blockade promotes neutrophil apoptosis, thus driving resolution of inflammation (Sousa et al., 2010). From a mechanistic point of view, we have recently discovered that selective blockade of PDE4 in human neutrophils downregulates Src family kinase activities (SFK)*,* through protein kinase A (PKA)–mediated activation of COOH-terminal Src kinase (CSK), a major endogenous regulator of SFK (Totani et al., 2014). Through these mechanisms, roflumilast, an oral selective PDE4 inhibitor approved for clinical use in patients with severe chronic obstructive pulmonary disease, prevents the release of NETs from neutrophils adhered on fibrinogen and challenged with bacterial endotoxin (Totani et al., 2016).

Here, we tested the hypothesis that PDE4 inhibitors may control NETosis in CF. The efficacy of PDE4 blockade was explored *in vitro*, using CF neutrophils, and *in vivo*, in a mouse model of *Pseudomonas aeruginosa* chronic lung infection.

## Materials and Methods

### Chemicals and Reagents

Selective PDE-4 inhibitors tested in this study were roflumilast-N-Oxide (RNO), the active metabolite of roflumilast (Bundschuh et al., 2001), kindly provided by Nycomed-Takeda (Konstanz, Germany), roflumilast for *in vivo* experiments by Aurogene (Rome, Italy), and rolipram by Calbiochem (Milan, Italy).

### Human Volunteers

Healthy donors were recruited among staff members of the Fondazione Mario Negri Sud. All donors signed an informed consent form. Individuals with CF (demographic characteristics are reported in Table 1) were recruited at the Cystic Fibrosis Center of Atri Hospital (TE, Italy) on an outpatient basis. Patients and their parents were informed about the rationale and objectives of the study and asked to sign the relative form. The experimental protocol was communicated to the Ethic Committee of the Institution, according to the national guidelines (G.U. n. 76 del 31–03–2008). Inclusion criteria: no antibiotics or steroids for at least 2 weeks before blood collection. Blood (15 ml) was collected in the occasion of a scheduled routine control. Pulmonary function was evaluated after a suspension of at least 12 h of bronchodilator or leukotriene receptor antagonist administration.

**TABLE 1:** Demographic characteristics of subjects with CF recruited for the study.

Age (Genotype)	FEV_1_%	Chronic infection	Discontinuous infection
27 years (G85E/R75X)	46,2	S.A.	P.A.
20 years (F508del/1303K)	38,1	S.A., P.A.	P.A.
17 years (UNK)	46,2	S.A., P.A., P.M., A.F.	A.F.
44 years (F508del/G542X)	25,3	B.C.	
40 years (F508del/G542X)	41,7	P.A.	
17 years (F508del/F508del)	92,0	S.A., P.A.	
22 years (F508del/F508del)	111,6	B.C.	S.A.

SA, Staphylococcus aureus; PA, Pseudomonas aeruginosa; BC, Burkholderia cenocepacia; PM, Proteus mirabilis; and AF, Aspergillus fumigatus.

### Neutrophil Isolation and Incubation

Neutrophils were isolated from the citrated whole blood by standard procedures routinely used in our laboratory (Evangelista et al., 2007). In order to mimic CFTR dysfunction, normal neutrophils were treated with CFTRinh-172 (10 µMol/L) for 15 min before experimental use. For adhesion, DMSO or CFTRinh-172–treated neutrophils (4 x 10^6^/ml), resuspended in HEPES-Tyrode buffer (pH 7.4) containing 129 mmol/L NaC1, 9.9 mmol/L NaHCO_3_, 2.8 mmol/L KCI, 0.8 mmol/L KH_2_PO_4_, 0.8 mmol/L MgCl-6H_2_O, 5.6 mmol/L Dextrose, 10 mmol/L HEPES, and 1 mmol/L CaC1_2_ were seeded on fibrinogen-coated (200 μg/ml; 200 μl/well for 24 h at 4°C) 12-well plates and allowed to adhere at 37°C, 5% CO_2_, in the absence or presence of bacterial endotoxin from *Escherichia coli*, serotype 055:B5 (Sigma-Aldrich, Milan, Italy) (10 μg/ml) for 18 h (Totani et al., 2016). In initial experiments, in order to set up the model, neutrophils were also stimulated by the classical chemoattractants fMLP (1 μMoles/L) and C5a (1 μMoles/L) or phorbol 12-myristate 13-acetate (PMA) (100 nMoles/L). When indicated, roflumilast-N-oxide (RNO) or a vehicle (DMSO) was added to cells 2 min before seeding.

### Neutrophil Extracellular Trap Measurements

DNA-NETs formed by neutrophils adherent to fibrinogen-coated surfaces were visualized by confocal microscopy after staining DNA with DRAQ-5 and FITC-conjugated anti-myeloperoxidase antibodies (Abcam, Cambridge, United Kingdom) (Totani et al., 2016). Extracellular DNA was quantified by a Quant-iT™ dsDNA high-sensitivity assay kit (Molecular Probes by Life Technologies, Oregon, United States) as previously described (Totani et al., 2016). Briefly, 2.5 µl of endonuclease (nuclease micrococcal, from *Staphylococcus aureus* 50 U/ml) (Sigma-Aldrich, Milan, Italy) was added to cell samples (500 µl) and incubated at 37°C for 10 min, followed by addition of 5 µl of EDTA (0.5 M) to stop the reaction. Samples were centrifuged at 10.000 x g in an Eppendorf centrifuge for 3 min, and supernatants stored at -20°C until DNA quantification.

### Citrullinated Histone-3 Detection

Pellets of BAL neutrophils (1 x 10^5^) were lysed with RIPA buffer. Pools of BAL supernatants or of cell lysates from all animals per each treatment group were analyzed by Western blotting using an anti-histone H3 (citrulline R2+R8+R17) (ab5103Abcam, Cambridge, Mass., United States).

### Immunoblotting

For Western blot analysis, the reaction was stopped by adding an equal volume of 2x reducing Laemmli’s lysis buffer, containing 2 mmol/L sodium orthovanadate, 5 mmol/L EGTA, 5 mmol/L EDTA, 10 mmol/L sodium pyrophosphate, 10 mmol/L iodoacetic acid, 1 mmol/L phenylmethylsulphonyl fluoride, 10 mmol/L sodium fluoride, 10 μg/ml leupeptin and aprotinin, and 1 mg/ml trypsin/chymotrypsin inhibitors, to the cell suspension. Samples were boiled for 10 min and centrifuged for 10 min at 10,000 g. Aliquots of 100 μl, corresponding to 0.2 x 10^6^ neutrophil total lysates (the supernatant and cells), were loaded into 10% gradient sodium dodecyl sulfate–polyacrylamide gel. Proteins were transferred onto nitrocellulose sheets, and nonspecific sites were blocked using 1% bovine serum albumin (BSA) in Tris-buffered saline overnight at room temperature on a horizontal shaker. The presence of citrullinated histone-3 was analyzed by immunoblotting with a rabbit, polyclonal, anti-histone H3 (citrulline R2+R8+R17) (ab5103Abcam, Cambridge, Mass., United States) (1:1,000; 1 h at room temperature) specific antibody, followed by incubation with an anti-rabbit ECL-conjugated secondary antibody (1:5,000; 1 h at room temperature). The ECL reagent (Perkin/Elmer, Inc) (MA/United States) was used for the detection of luminescence, by UVITEK.

### Flow Cytometry

Neutrophils were analyzed by flow cytometry to quantify the percentage of neutrophils: 1) remaining morphologically intact, 2) showing markers of apoptosis, and 3) containing myeloperoxidase, after 18 h of adhesion to fibrinogen-coated surfaces. To this end, neutrophils were detached from fibrinogen by a brief exposure to EDTA/EGTA (both 5 mMol/L). Neutrophil apoptosis was quantified by a FITC-conjugated annexin-V/propidium iodide (P.I.) kit according to the manufacturer’s instruction (BD-Biosciences, United States). The intracellular myeloperoxidase content was measured by the “MPO-FITC” Kit according to the manufacturer’s instruction (Beckman Coulter, Milan, Italy).

### Animal Studies

Animal studies adhered strictly to the Italian Ministry of Health guidelines for the use and care of experimental animals (protocol #549 and 733). Research with *P. aeruginosa* RP73 isolate from CF individuals has been approved by the Ethics Commission of Hannover Medical School, Germany. The patient and parents gave informed consent before the sample collection. Approval for storing biological materials was obtained by the Ethics Commission of Hannover Medical School, Germany. C57Bl/6NCrlBR male mice (8–10 weeks of age) from Charles River Laboratories were challenged with 1 × 10^6^ CFU of MDR-RP73 embedded in agar beads for chronic infection by intratracheal administration, as previously described (Bragonzi, 2010; Paroni et al., 2013; Facchini et al., 2014).

First, mice were treated by gavage with roflumilast (5 mg/kg) or a vehicle (4.4%DMSO in saline) daily, starting 2 h before infection. Health and body weight were monitored daily. Mice were killed 5 days after infection, 2 h after the last treatment. The lungs were excised and analyzed for bacterial loads, by measuring the colony forming units (CFUs). The bronchoalveolar lavage fluid (BALF) was analyzed for the total and differential cell count, amount of free DNA, and cytokine content. Free DNA was measured by a Quant-iT™ dsDNA high-sensitivity assay kit as followed for *in vitro* experiments. Cytokines were measured using a competitive ELISA method or a Luminex multi-analyte assay (ProcartaPlex, Thermo Fisher Scientific, Monza, Italy).

Next, mice were treated per aerosol with roflumilast (5 mg/kg) or a vehicle (4,4% DMSO in saline) using Penn Century as previously described (Cutone et al., 2019). The drug or vehicle was administered once a day, starting from 4 h after infection. Each group of treatment was divided into two: one group of animals was killed 28 h after infection (2 h after treatment) to analyze the effect of treatments on the acute phase of the infection, whereas the other group was killed 5 days after infection (i.e., 2 h after the last treatment) to analyze the effect of treatments in chronic infection.

Body weight was determined, and aerosol administration was carried out once a day under anesthesia (5% isoflurane–oxygen, running at 4 l/min) according to established procedures. At the end of the experiment, the BALF was collected and analyzed for cells, free DNA, and cytokine content. A fraction of the BALF containing a fixed number of neutrophils (1 x 10^5^) was centrifuged, and the pelleted cells were immediately frozen and stored for Western blot analysis of citrullinated histone H3. The lungs were excised and homogenized, and CFU counting was performed as reported (Bragonzi, 2010; Paroni et al., 2013; Facchini et al., 2014; Cutone et al., 2019).

### Statistical Analysis

Data from *in vitro* experiments were reported as the mean and standard deviation. The paired *t*-test was used to analyze the differences between treated and untreated cell samples. An analysis-of-variance (ANOVA), accounting for correlation within matched pairs, was used to explore concentration–response relationships. *p*-values < 0.05 were considered statistically significant. Data from *in vivo* experiments are reported as box plots: the horizontal line in the middle of the box marks the median of values; the edges of each box mark the 25th and 75th percentiles; and vertical lines, extending up and down from each box, represent largest and smallest values, respectively, that are not outliers (values greater than 1.5 times the length of the box were considered outliers and excluded from the analysis). Paired *t*-test was used analyze the differences between treated and untreated groups. *p*-values < 0.05 were considered statistically significant.

## Results

### PDE4 Blockade Prevents Neutrophil Extracellular Trap Release

**FIGURE 1:**
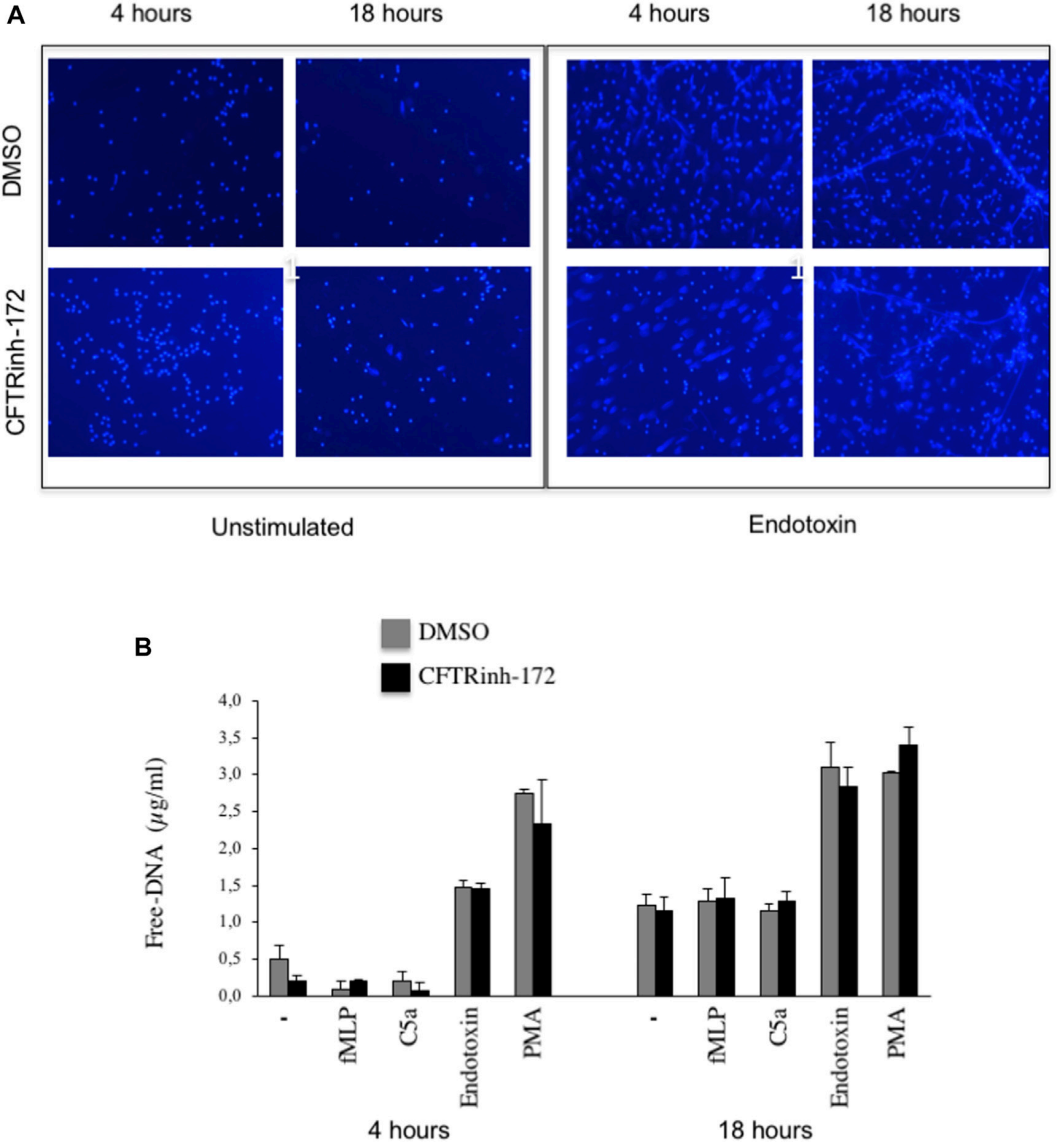
**(A)** Neutrophils isolated from healthy subjects were allowed to adhere on fibrinogen-coated slides in the absence or presence of endotoxin (10 μg/ml) and cultured for 4 or 18 h. At the end of the incubation, samples were fixed and stained for DNA (blue staining, DRAQ-5). **(B)** Neutrophils from healthy subjects pretreated with CFTRInh-172 (10 µMoles/L) or a vehicle were allowed to adhere on fibrinogen-coated surfaces and stimulated with different agonists for 4 or 18 h. At the end of the incubation, free DNA was quantitated. Briefly, 2.5 µl of endonuclease (nuclease micrococcal, from *Staphylococcus aureus* 50 U/ml) per 500 µl of the sample was added, and samples were incubated at 37°C for 10 min. The reaction was stopped with 5 µl of EDTA (0.5 M). Samples were centrifuged at 10.000 x g for 3 min, and supernatants were stored at -20°C until DNA quantification by Quant-iT™ dsDNA high-sensitivity assay kit (Invitrogen). Results are means ± SEM of experiments with cells from 4 different donors performed in duplicates.

To analyze NET release *in vitro*, we stimulated neutrophils, adhered on fibrinogen-coated surfaces, with bacterial endotoxin from *Escherichia coli* at 37°C, 5% CO_2_. NETs were visualized by confocal microscopy after DNA staining. After 4 h of stimulation with endotoxin, most of the neutrophils showed decondensation of their nuclei, and DNA appeared widespread into the cytoplasm and outside the cell (Figure 1A **)**. Few extracellular DNA filaments were visible at this early time point. After 18 h, macroscopic NET structures (Figure 1A) were diffused among cells. In order to explore the potential role of CFTR in NETosis, we evaluated, in parallel, NET formation in neutrophils that were untreated or exposed to CFTRinh-172, a selective CFTR inhibitor. As shown in Figure 1A, NET formation was not apparently influenced by CFTR inhibition. For a more quantitative analysis, extracellular free DNA was measured in supernatants. Released DNA was barely detectable after 4 h in unstimulated cells, and it was not significantly enhanced by the classical chemoattractants fMLP and C5a. A substantial increment was instead observed in the presence of endotoxin or PMA (Figure 1B)**.** A similar pattern was observed after 18 h of incubation (Figure 1B). Free-DNA quantitation confirmed that CFTR inhibition did not significantly influence NET release (Figure 1B).

**FIGURE 2:**
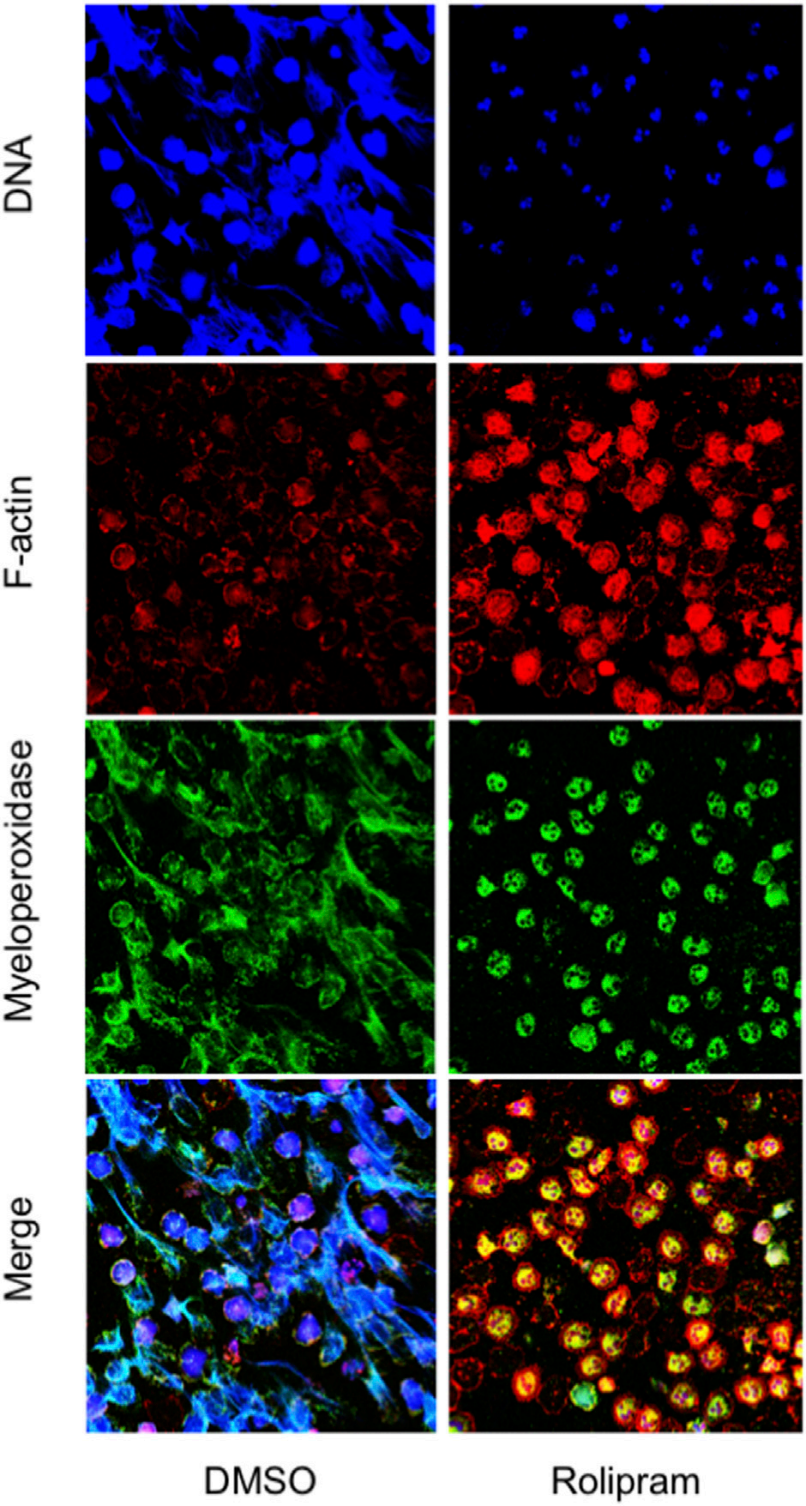
Neutrophils isolated from healthy subjects were allowed to adhere on fibrinogen-coated slides in the presence of endotoxin, with or without rolipram (10 µMoles/L) and cultured for 18 h. At the end of the incubation, samples were fixed and stained for DNA (blue staining, DRAQ-5), myeloperoxidase (green staining, FITC-conjugated anti-myeloperoxidase antibody), and F-actin (red staining, TRIC-phalloidin) and analyzed by confocal microscopy. The figure shows images representative of three different experiments.

**FIGURE 3:**
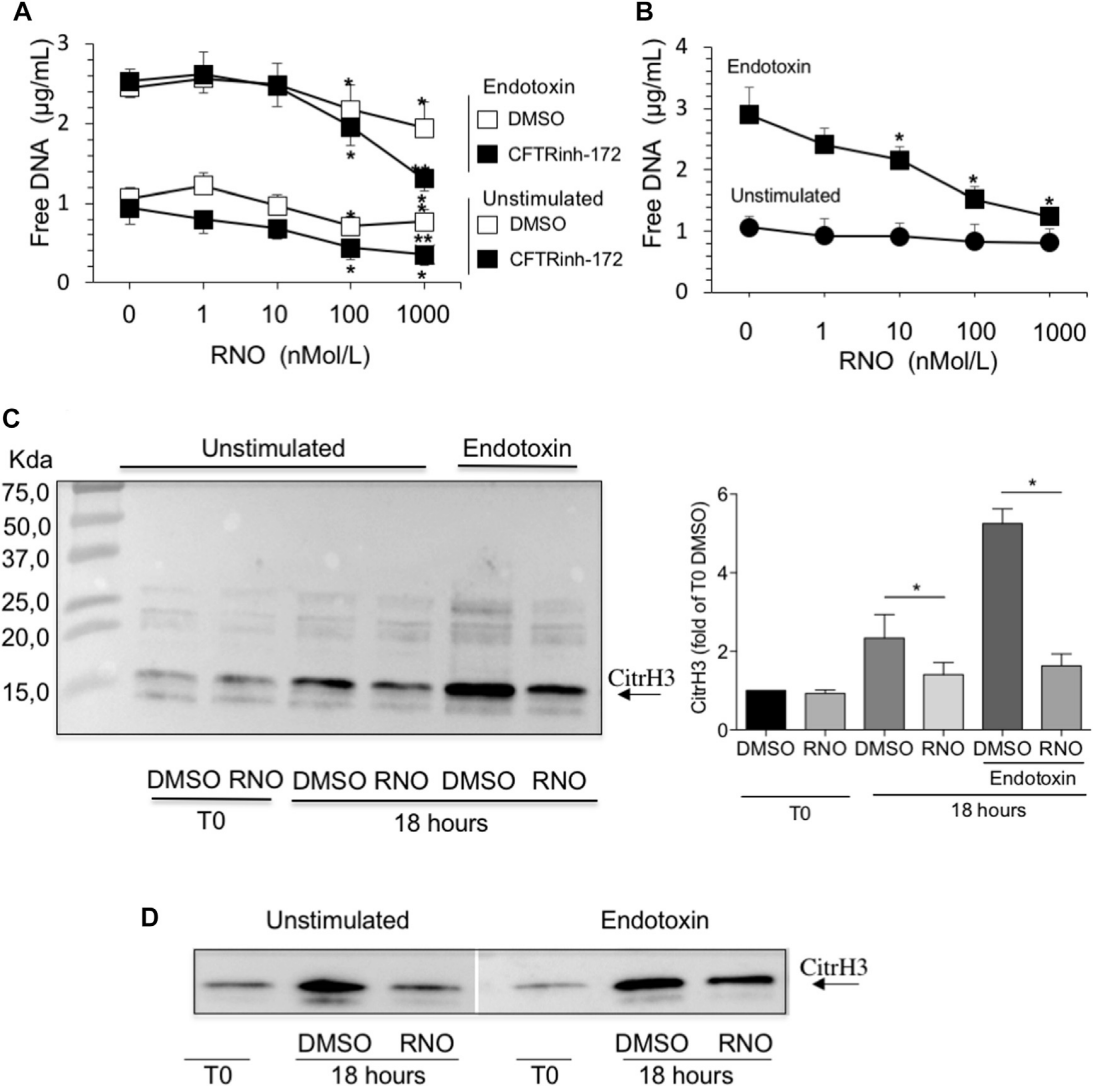
**(A)** Neutrophils from healthy subjects, pretreated for 2 min with increasing concentrations of RNO (0–1,000 nMoles/L), were exposed to endotoxin and allowed to adhere on fibrinogen-coated surfaces for 18 h in the presence or in the absence of CFTRinh-172 (10 µMoles/L). Unstimulated neutrophils, pretreated with increasing concentrations of RNO (0–1,000 nMoles/L), were incubated in parallel. At the end of the incubation, free DNA was quantitated. Results are mean ± SEM of experiments performed with cells from 9–11 different donors in duplicates. *p < 0.05 (ANOVA, Dunnett test), RNO-treated vs untreated samples; **p < 0.05 (Student’s *t*-test), CFTRinh-172–treated vs untreated samples. **(B)** Neutrophils from individuals with CF, pretreated with increasing concentrations of RNO (0–1,000 nMoles/L), were incubated with endotoxin and allowed to adhere on fibrinogen-coated surfaces for 18 h. Unstimulated neutrophils, pretreated with increasing concentrations of RNO (0–1,000 nMoles/L), were incubated in parallel. Results are mean ± SEM of experiments performed with cells from seven different patients with CF (see Table 1 for patients’ characteristics). *p < 0.05 (ANOVA, Dunnett test) vs untreated samples. The presence of citrullinated histone H3 in neutrophils from three healthy donors **(C)** or two people with CF **(D)** was analyzed after 18 h of incubation. Samples were then subjected to Western blot analysis using a monoclonal antibody which specifically recognizes citrullinated histone H3. Representative Western blots are shown. The right image in panel C reports a densitometric analysis from n = 3. *p < 0.05 (Student’s t-test).

Next, we examined the impact of PDE4 blockade on neutrophil morphology and DNA release by confocal microscopy and free-DNA quantitation. NET structures were visualized by DNA and myeloperoxidase staining with DRAQ-5 and an FITC-conjugated anti-myeloperoxidase antibody, respectively. After 18 h of incubation, DNA filaments decorated with myeloperoxidase were diffused among cells. Notably, rolipram (10 μM) preserved morphological integrity, nuclear DNA, and cytoplasmic myeloperoxidase localization (Figure 2). Consistently, RNO (the active metabolite of roflumilast) concentration-dependently (0.1–1 Mol/L) reduced DNA release by neutrophils from healthy volunteers as this effect was more evident in the presence of CFTRinh-172 (Figure 3A). Similar measurements were carried out with neutrophils isolated from seven patients with CF. As shown in Figure 3B, RNO concentration-dependently reduced free-DNA release.

NET formation requires the activation of peptidylarginine deiminase 4 (PAD4) that converts arginine to citrulline on nuclear histones and promotes chromatin decondensation. Thus, histone H3 citrullination may be considered a specific NETosis marker (Wang et al., 2009; Papayannopoulos et al., 2010; Thiam et al., 2020). To unequivocally confirm that reduction of free DNA was a consequence of inhibition of NETosis, we assessed the presence of citrullinated histone H3 in cell lysates from neutrophils isolated from healthy volunteers and people with CF by Western blot analysis. Figure 3 shows that RNO reduced citrullination of histone H3, demonstrating that PDE4 blockade controls biochemical events necessary for NET formation in healthy (Figure 3C) and CF (Figure 3D) neutrophils.

### PDE4 Blockade Preserves Neutrophil Integrity and Apoptosis

**FIGURE 4:**
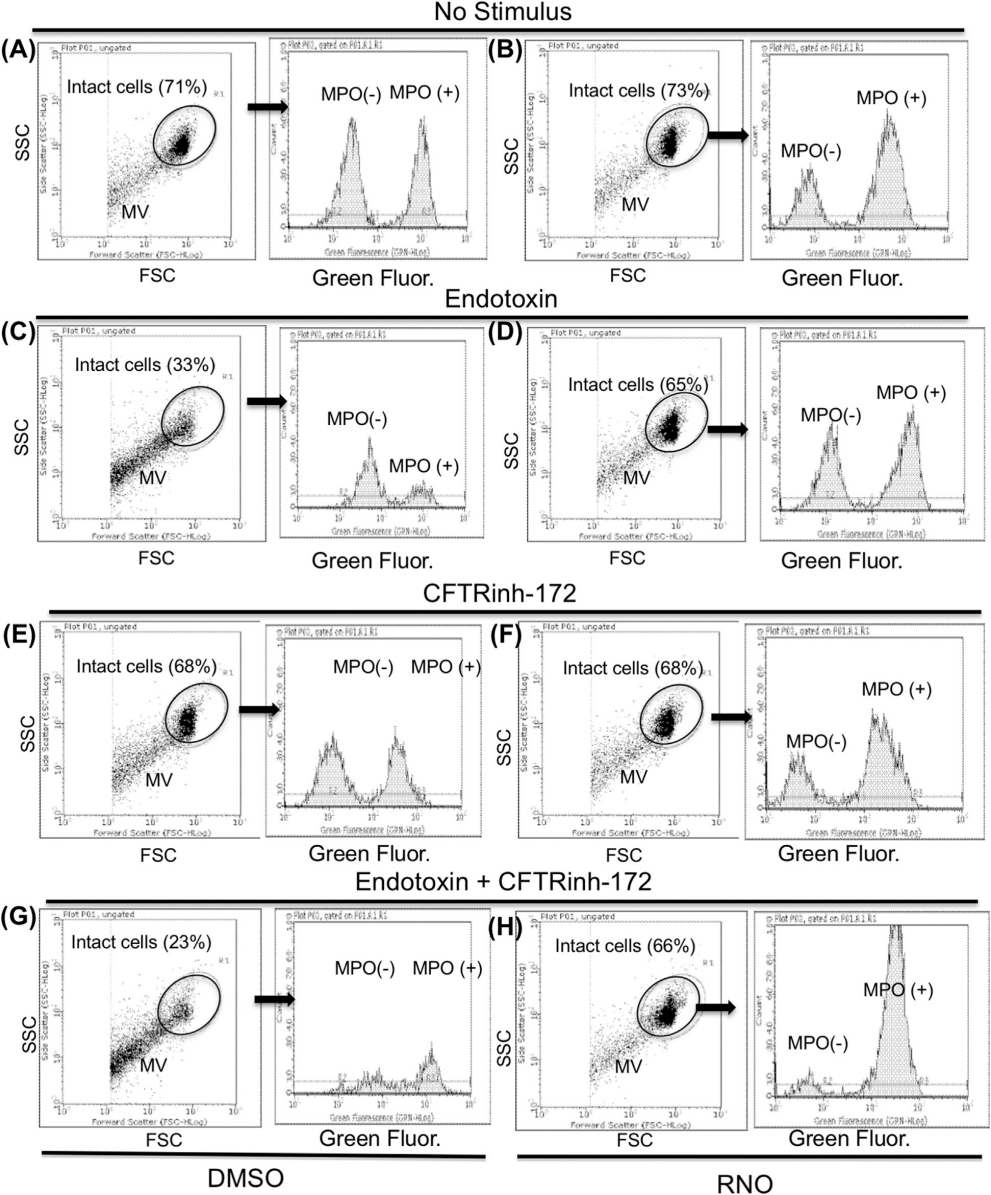
Neutrophils from healthy subjects pretreated with a vehicle **(A,C,E,G)** or RNO (100 nMoles/L) **(B,D,F,H)** were exposed to endotoxin and allowed to adhere on fibrinogen-coated surfaces for 18 h in the absence **(C,D)** or in the presence **(G,H)** of CFTRinh-172 (10 µMoles/L). Unstimulated neutrophils were incubated in parallel in the absence **(A,B)** or in the presence **(E,F)** of CFTRinh-172 (10 µMoles/L). At the end of the incubation, samples were stained for intracellular myeloperoxidase and analyzed by flow cytometry. Intact neutrophils were identified on the basis of typical SSC and FSC and analyzed for myeloperoxidase content. Results are from one representative experiment MV (neutrophil microvesicles).

**FIGURE 5:**
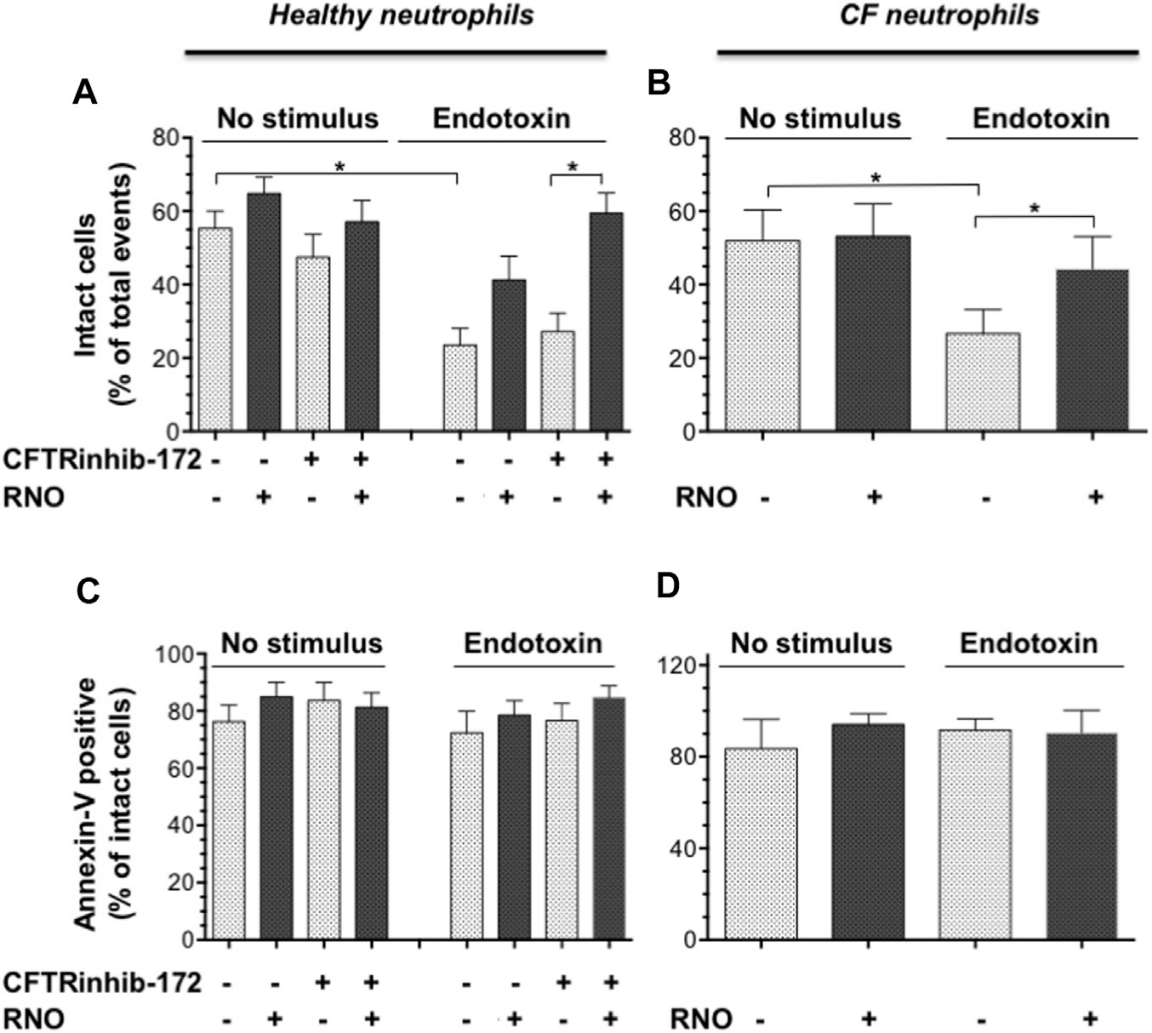
Neutrophils isolated from healthy subjects (n = 16) **(A,C)** or from volunteers with CF (n = 7) **(B,D)** were treated with RNO (100 nMoles/L) (shaded bars) or a vehicle (white bars) and allowed to adhere on fibrinogen-coated surfaces in the presence or absence of CFTRinh-172 (10 μMoles/L), for 18 h with or without endotoxin. At the end of the incubation, the percentage of intact cells **(A,B)** and annexin V binding related to intact cells **(C,D)** was evaluated by flow cytometry (Figure 4). Intact neutrophils were identified by typical SSC and FSC. *p < 0.05 (Student’s *t* test) vs vehicle-treated samples.

NETosis is accompanied by microvesiculation and fragmentation of netting neutrophils (Thiam et al., 2020). Images shown in Figure 2 are suggestive of protective effects by the PDE4 inhibitor on neutrophil fragmentation. To obtain a more quantitative readout of this effect, we used flow cytometry. To this end, after 18 h of adhesion, neutrophils from normal volunteers were detached from fibrinogen-coated surfaces by a brief exposure to 5 mMol/L EGTA/EDTA, permeabilized, and stained with a FITC-conjugated anti-myeloperoxidase antibody. Figure 4 reports a flow cytometric analysis showing that, compared to unstimulated samples (A), endotoxin stimulation (C) significantly decreased the percentage of intact neutrophils. Moreover, in the absence of endotoxin, 50% of the intact neutrophil population contained large amounts of intracellular myeloperoxidase (A), indicating the presence of nondegranulated cells. In contrast, in endotoxin-treated samples, the few neutrophils remaining intact appeared completely degranulated (C). No significant changes were detected in the presence of CFTRinh-172 (Figures 4E and G, without or with endotoxin, respectively). PDE4 inhibition by RNO (100 nMoles/L) significantly increased the percentage of intact neutrophils as well as of non-degranulated cells in all experimental settings (Figures 4B,D,F, and H, and Figure 5A). These effects were quantitated in neutrophils from seven donors with CF and 16 healthy volunteers. We consistently observed that RNO (100 nMoles/L) increased the percentage of intact neutrophils in endotoxin-stimulated samples (Figures 5A,B). Since neutrophil apoptosis is key for the proper development of the resolution program of the inflammatory response and neutrophils from patients with CF manifest delayed apoptosis (McKeon et al., 2008; Moriceau et al., 2010; Gray et al., 2018), we asked whether preservation of neutrophil integrity by PDE4 inhibition had an impact on neutrophil apoptosis. For this purpose, neutrophils were stained with annexin V and P.I. to assess the percentage of early/late apoptotic or necrotic cells. Results reported in Supplementary Tables S1,S2 clearly show that the majority of intact neutrophils, both healthy and CF, were annexin V positive/P.I negative; a lower number showed annexin V positive/P.I. positive. In contrast, the fraction of viable (annexin V negative/P.I. negative) or necrotic cells (annexin V negative/P.I. positive) was negligible. Figures 5C and D summarize that approximately 80% of endotoxin-stimulated normal (±CFTRinh-172) or CF neutrophils, which remained intact after 18 h, displayed an apoptotic profile. Collectively, these results suggest that PDE4 inhibition controlling NETosis and, at the same time, preserving neutrophil apoptosis may be useful to mitigate neutrophilic inflammation in CF. To further support this hypothesis, we conducted preclinical studies in a mouse model of bacterial lung inflammation.

### Effect of PDE4 Inhibition on Lung Inflammation and Infection in Mice

**FIGURE 6:**
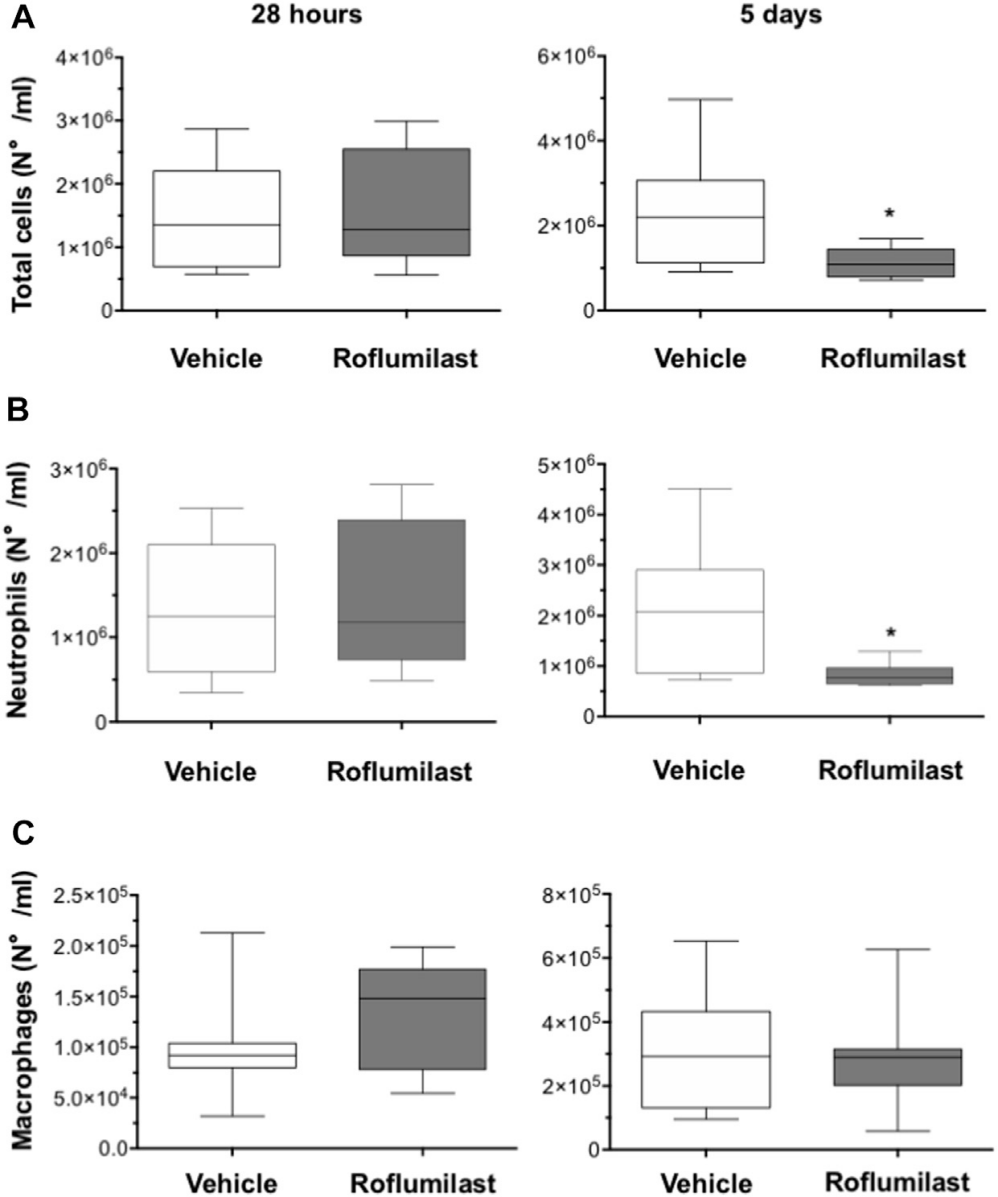
C57BL/6 male mice (8–10 weeks of age) were infected i.t. with 1 × 10^6^ CFUs of MDR-RP73 embedded in agar beads and *per aerosol* with roflumilast (5 mg/kg) or placebo (4,4% DMSO in saline) once a day starting from 4 h after infection. Animals were killed after 28 h or 5 days of infection, and the BALF was collected. Total cells **(A)**, neutrophils **(B),** and macrophages **(C)** were counted in the BALF. Panels show box plots of cell numbers at the time of killing(28 h and 5 days after infection) of vehicle- (n = 9 per group) or roflumilast-treated mice (n = 9 killed at 28 h and n = 8 killed at 5 days after infection). The horizontal lines mark the median of values, the edges of each box mark the 25th and 75th percentiles, and the vertical lines indicate the highest and lowest values, respectively, which are not outliers (values greater than 1.5 times the length of the box were considered outliers and excluded from the analysis). *p < 0.05 (ANOVA, Dunnett’s test) *vs* vehicle-treated mice.

**FIGURE 7:**
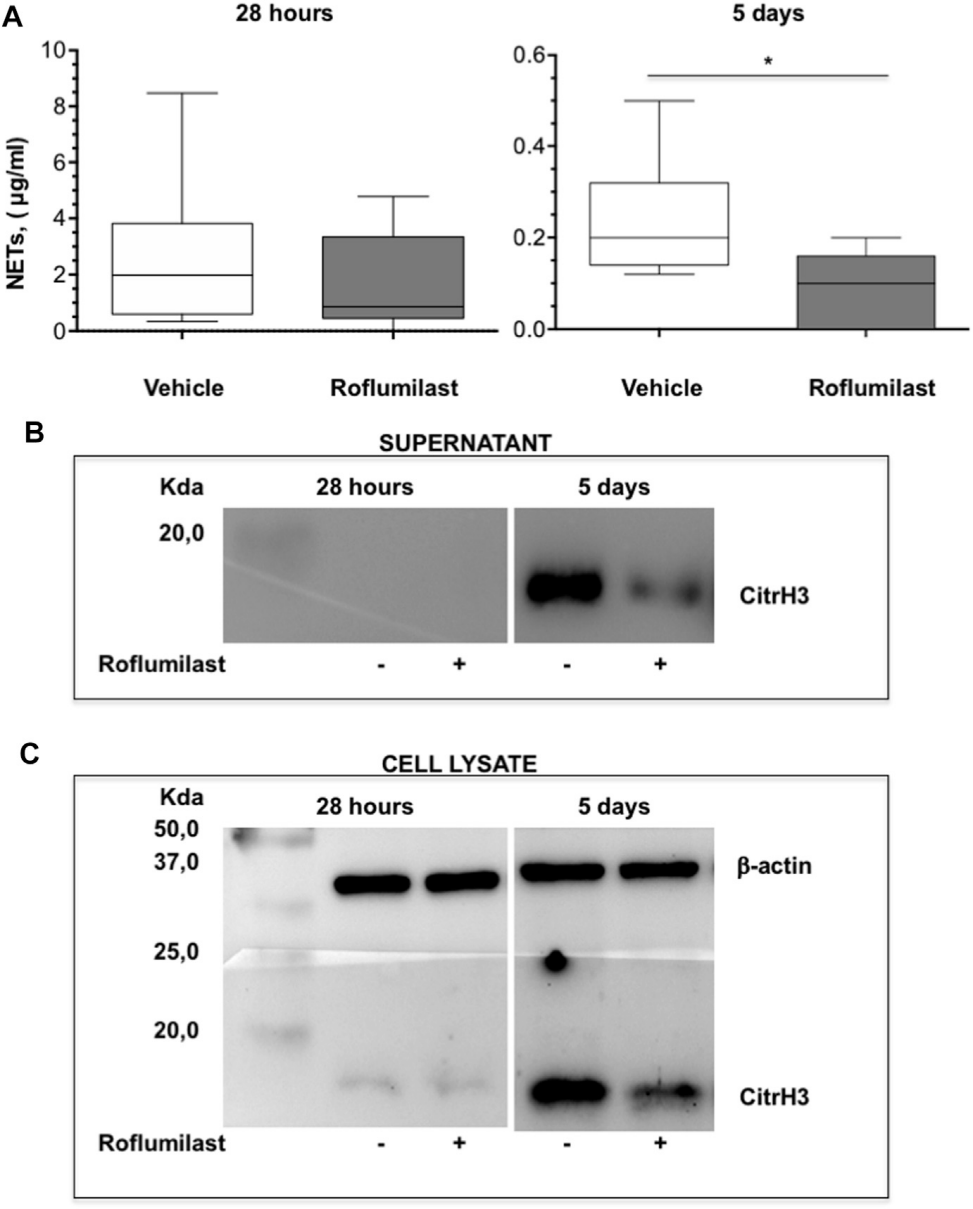
C57BL/6 male mice were treated as described in Figure 6 and killed after 28 h or 5 days of infection. The BALF was collected for measurement of free DNA in supernatants and citrullinated histone H3 in supernatants and in cell lysates. **(A)** shows box plots of free-DNA values at the time of killing (28 h and 5 days after infection) of vehicle- (n = 9 per group) or roflumilast-treated mice (n = 9 killed at 28 h and n = 8 killed at 5 days after infection). *p < 0.05 (ANOVA, Dunnett’s test) *vs* vehicle-treated mice. The presence of citrullinated histone H3 was analyzed by Western blotting in BALF supernatants **(B)** as well as in lysates of BALF cells from mice killed at 28 h and 5 days after infection **(C)**. Pools of BALF supernatants and of cell lysates from all animals of each group were subjected to Western blotting using a monoclonal antibody which recognizes mouse with citrullinated histone H3.

**FIGURE 8:**
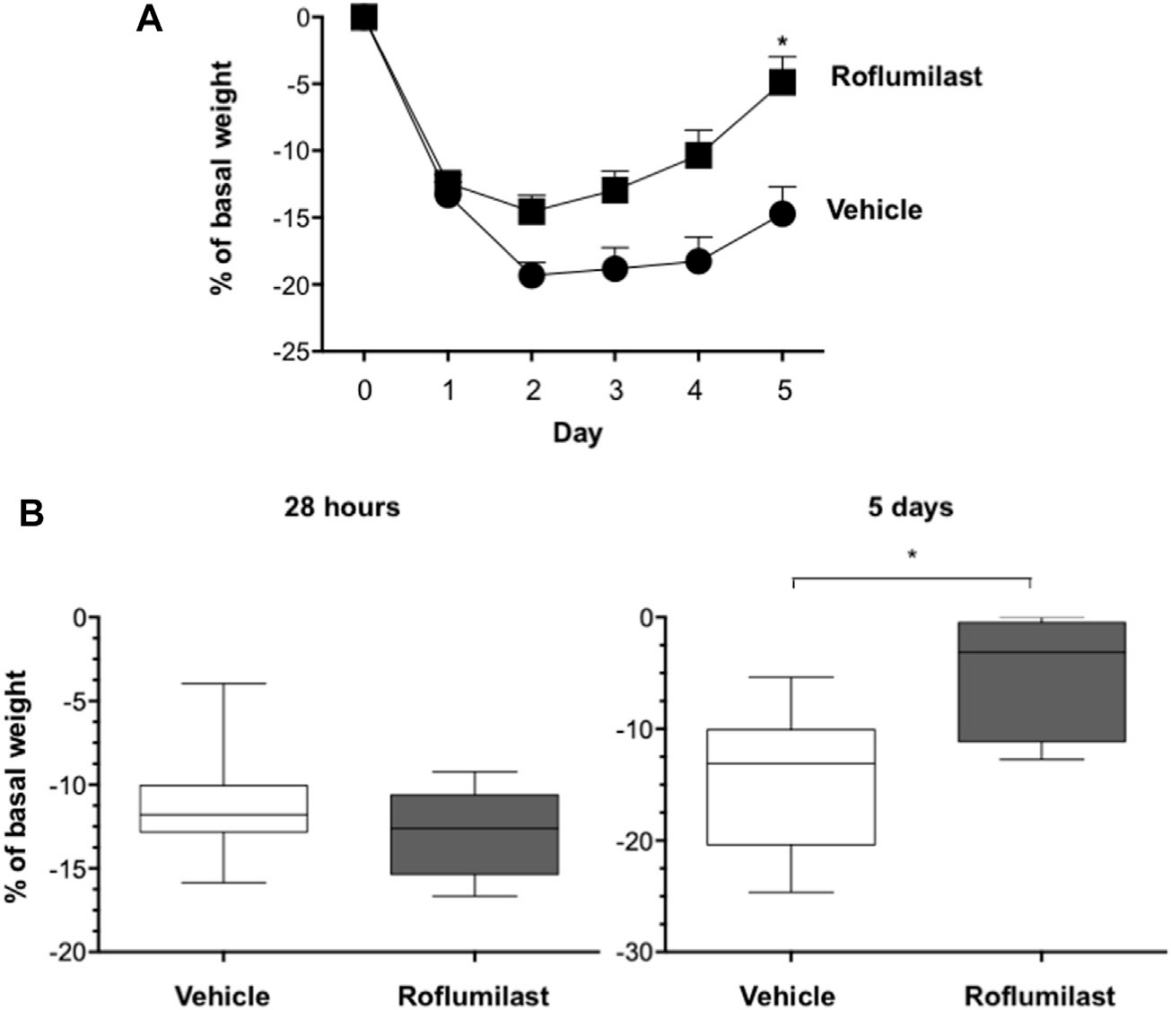
C57BL/6 male mice were treated as described in Figure 6. **(A)**. Body weight of mice was monitored daily before treatment to evaluate the health status. *p < 0.05 (ANOVA, Dunnett test) *vs* vehicle-treated mice. **(B)** shows box plots of weight loss values at the time of killing(28 h and 5 days after infection) of vehicle- or roflumilast-treated mice. *p < 0.05 (Student’s t-test) *vs* vehicle-treated mice.

First, the efficacy of oral administration of roflumilast was tested in a murine model of *Pseudomonas aeruginosa* infection to mimic a chronic lung infection similar to the one typically established in the airways of people with CF. C57Bl/6NCrlBR mice were challenged with 1 x 10^6^ MDR-RP73 embedded in agar beads by intratracheal administration to induce chronic infection. Mice were treated with roflumilast (5 mg/kg) or a vehicle (4,4% DMSO in saline) by gavage once a day for 5 days, starting 2 h before infection. Body weight and health of mice were monitored daily. After 5 days of infection (2 h after the last treatment), mice were killed, and the BALF was collected and analyzed for the total and differential cell count, protein content, as markers of vascular permeability, and interleukin (IL)-1β, tumor necrosis factor (TNF)-α, and KC, the analogue of IL-8 in the mouse, levels as indices of inflammation. The amount of free DNA in the BALF was analyzed as an indirect measurement of NETs. In addition, since treatments that impair neutrophil activities may potentially reduce immune responses to bacterial infection, CFUs were counted in BAL and in homogenized lung tissue. The effects of oral administration of roflumilast on bacterial loads and inflammatory (cells and cytokines) markers in the BALF are summarized in Table 2. No difference was observed in the lung CFU after 5 days of infection between roflumilast- and vehicle-treated animals. We observed a clear trend toward a reduction in the inflammatory response in the roflumilast-treated group compared to the vehicle-treated group. Statistically significant differences were detected in total cells and in the number of neutrophils, but no differences were observed in the number of macrophages. Roflumilast treatment induced a statistically significant reduction in TNF-α, while the reduction in KC and IL-1β levels was not significant. Free DNA and total protein content also appeared to be reduced in the BALF of treated mice, but the differences did not reach the statistical significance. Based on these results, we reasoned that, compared with the oral route, intratracheal administration could yield higher drug concentration in the airways and more effectively control neutrophil recruitment and activation. Three doses of roflumilast (0.5, 1, or 5 mg/kg/day) were tested for toxicity in noninfected animals. No evidence of side effects was recorded for all doses (data not shown). Therefore, we explored the efficacy of intratracheal administration of roflumilast (5 mg/kg/day for 5 days) in C57Bl/6NCrlBR mice infected with *Pseudomonas aeruginosa* MDR-RP73 embedded in agar beads*.* To exclude possible effects of the drug during the initial phase of bacterial infection, the first treatment was started 4 h after infection. For each group of treatment, a half number of animals were killed 28 h after the infection, 2 h after the second treatment, to analyze the effect of the drug in the acute phase, and the remaining were killed 5 days after infection, 2 h after the last treatment. At these time points, the BALF and lungs were collected for analyses. We observed that the number of total cells, neutrophils, and macrophages, increased at 5 days with respect to 28 h, in the vehicle-treated group (Figures 6A–C), indicating persistence of cell recruitment. Treatment with roflumilast, which did not modify inflammatory cells in the BALF at 28 h of infection, significantly reduced the number of total cells and the number of neutrophils at 5 days post infection compared to vehicle-treated animals (Figures 6A,B). On the contrary, the macrophage count was not affected by roflumilast (Figure 6C). Moreover, roflumilast-treated mice showed significantly lower amount of free DNA in the BALF at 5 days, compared with vehicle-treated animals (Figure 7A). To unequivocally confirm that the reduction in free DNA was a consequence of NETosis inhibition, we analyzed the presence of citrullinated histone H3 in supernatants and lysates from neutrophils recovered in the BALF. To this purpose, pools of supernatants and cell lysates from all BALFs of each group were subjected to the Western blot analysis of citrullinated histone H3. As shown in Figures 7B and C, citrullinated histone H3, which was undetectable in samples collected after 28 h from infection, increased both in supernatants and inside the cells in samples collected 5 days after infection. At this time point, BALF supernatants and cells collected from animals treated with roflumilast displayed reduced citrullinated histone H3 compared to vehicle-treated mice, conclusively demonstrating that PDE4 inhibition controls DNA release in inflamed airways by blocking biochemical events necessary for NET formation. Measurements of body weight, as an index of the general health status of infected animals, indicated a rapid decrease in body weight in both groups of treatment. The group of mice treated with roflumilast recovered weight more rapidly than the vehicle-treated group (Figure 8A). The improvement in recovery was statistically significant 5 days after infection (Figure 8B). The bacterial load in the BALF and lungs at 28 h and 5 days was not affected by roflumilast treatment and decreased by approximately an order of magnitude 5 days after infection compared to the 28-h time point, in all groups of treatment (Supplementary Figure S1). Likewise, the amounts of KC, TNF-α, and MIP2 decreased at 5 days and were not significantly affected by roflumilast (Supplementary Figure S2).

**TABLE 2:** Effect of oral administration of roflumilast on CFUs in the lungs and inflammatory markers in the BALF.

	Vehicle	Roflumilast
Total cells (10^6^/ml)	2,49 ± 2,1	1,57 ± 1,2 (*p* = 0,04)
Neutrophils (10^6^/ml)	1,84 ± 1,0	1,04 ± 1,14 (*p* = 0,05)
Macrophages (10^6^/ml)	0,52 ± 0,2	0,48 ± 0,4 (*p* = 0,41)
Interleukin-1β (pg/ml)	114,3 ± 51,2	80,27 ± 69,0 (*p* = 0,10)
TNF-α (pg/ml)	1506,9 ± 263,9	1184,4 ± 392,1 (*p* = 0.008)
CXCL-1 (KC) (pg/ml)	19,0 ± 9,0	13,7 ± 11,1 (*p* = 0,12)
Free DNA (μ/ml)	2,2 ± 2,3	1,5 ± 1,4 (*p* = 0,19)
CFU - lung (x 1,000)	49,6 ± 52,9	55,9 ± 91,7 (*p* = 0,47)
Total proteins (mg/ml)	558,7 ± 208,3	445,8 ± 119,3 (*p* = 0,07)

C57BL/6 male mice (8–10 weeks of age) were infected i.t. with 1 × 10^6^ CFUs of MDR-RP73 embedded in agar beads. Mice (11 per group of treatment) were treated by gavage with roflumilast (5 mg/kg/day) or placebo (4,4% DMSO in saline), with the starting dose administered 2 h before infection and the last 2 h before killing. 5 days after infection, mice were sacrificed, the BALF was collected, and mouse lungs were recovered, homogenized, and plated to determine the bacterial load. Counts of total cells, neutrophils, and macrophages, as well as levels of cytokines and total protein, were evaluated in the BALF. Statistical differences between roflumilast- and vehicle-treated animals were analyzed by Student’s t-test.

Correlation analyses between inflammation markers and the body weight loss at 5 days after infection, including all animals treated with roflumilast or a vehicle of both experimental protocols (*per os* or *intratracheal*) (Supplementary Figure S3), showed that the number of neutrophils **(**A) as well as the amount of free DNA (B), KC (D), and total proteins (F) in the BALF, positively correlated with the weight loss. In contrast, no or very weak correlations were found between the number of macrophages (e), the amount of IL-1β (G), TNF-α (C), and weight loss. Unexpectedly, the bacterial load (the CFU in homogenates of lung tissue) did not correlate with the weight loss (H).

## Discussion

The mechanisms of neutrophilic inflammation in CF lung disease remain to be fully understood and no established pharmacological treatments are currently available to control this process. However, recent studies strongly suggest that NETs are main determinants of lung inflammation and damage in CF. Therefore, identifying therapies that preserve the positive effects of neutrophils, while reducing the detrimental effects of NETs and cytotoxic components, is essential for achieving innovative therapeutic advances (Khan et al., 2019). Here, we provide evidence that PDE4 inhibition prevents NETosis in, *in vitro* and *in vivo*, CF-relevant models.

In this work, we tested the selective PDE4 inhibitor roflumilast mainly because this drug has been tested in phase II and III clinical trials (Rabe, 2011) and has been approved as an adjuvant to reduce the risk of exacerbation in patients with severe COPD (Schudt et al., 2011). Like CF, COPD is, in fact, characterized by excessive neutrophilic lung infiltration as well as by an imbalance in the oxidant/antioxidant and protease/antiprotease equilibrium, two neutrophil-mediated processes, regarded as major determinants of the progressive lung damage in both diseases. PDE4 blockade in lung immune cells prevents the progression of inflammation in COPD (Rabe, 2011; Baye, 2012). Moreover, roflumilast reduced neutrophil and eosinophil accumulation in BAL of healthy volunteers subjected to segmental administration of endotoxin (Hohlfeld et al., 2008). Further indication for the clinical use of PDE4 inhibitors in CF derives from the evidence that PDE4 inhibition rescues CFTR activity in varying experimental settings (Blanchard et al., 2014).

RNO reduced NET release by neutrophils *in vitro* (Figures 1–3). This was clearly demonstrated by combining confocal microscopy with measurements of free DNA and citrullinated histone H3. These analyses were conducted on neutrophils from healthy donors, exposed or not exposed to CFTRinh-172 to mimic a CF status, and on neutrophils from donors with CF with different CFTR mutations and disease severity (Table1). The overall picture emerging from these experiments shows that while CFTR blockade did not modify the entity of NET release, RNO was significantly more potent at inhibiting NETosis in CFTRinh-172–treated normal neutrophils as well as in CF neutrophils, compared to untreated normal neutrophils. While confirming previous data from our and other’s laboratory (Shishikura et al., 2016; Totani et al., 2016), the present results provide the novel observation that PDE4 blockade is more efficient at reducing NETs under CF conditions, suggesting that impairment of CFTR function induces signaling events that favor the activity of the PDE4 inhibitor.

In addition to reducing NETs, RNO preserved neutrophil integrity as well as the stability of MPO-containing intracellular granules (Figure 4). These are key events in neutrophil-driven inflammation since the release of the content of proteolytic enzymes in the airways, stored in neutrophil granules, sustains CF inflammation and lung damage. Along these lines, the observation that RNO preserves both neutrophil integrity and the apoptotic process further supports the hypothesis that PDE4 inhibition may sustain the resolution program of inflammation, which requires a discrete neutrophil apoptosis to be preserved. A recent work links NETosis with a delayed neutrophil apoptosis in CF and suggests that promotion of apoptosis may allow more appropriate neutrophil disposal, decreasing NET formation and inflammation (Gray et al., 2018). The existence of a functional link between the two processes, NETosis and apoptosis, is also supported by the results of Remijsen et al. (2011) showing that inhibition of either autophagy or NADPH oxidase activity, which are essential for NET formation, results in cell death characterized by hallmarks of apoptosis, suggesting that switching-on an apoptosis program might function as a stop signal for NETosis and *vice versa*. In keeping with this, our results in *in vitro* models demonstrate that PDE4 blockade reduces NETosis while preserving apoptosis, and this is particularly relevant in CF neutrophils. Biochemical pathways mediated by cAMP-activated PKA and regulating kinases belonging to the src family as well as the PI3K/Akt pathway appear to play a relevant role in this effect (Sousa et al., 2010; Totani et al., 2014, 2016).

*In vitro* data were confirmed in a preclinical mouse model of respiratory infection by *Pseudomonas aeruginosa*, which often colonizes the airways of patients with CF. We used two different routes of roflumilast administration, by gavage or intratracheal, and monitored markers of neutrophilic inflammation at short (28 h) and extended time (5 days). The results showed that while these markers were barely affected by the drug given *per os*, the aerosol administration was effective in reducing the accumulation of neutrophils at 5 days but not after 28 h of infection. Accordingly, roflumilast-treated mice showed a significant reduction in the accumulation of free DNA in the BALF at 5 days, as well as reduced citrullination of histone H3, both in BALF supernatants and cells, thus conclusively demonstrating that PDE4 inhibition controls key biochemical steps necessary for NET formation *in vivo*. Of note, roflumilast did not modify DNA release after 28 h of infection, and at this early time point, we did not observe measurable amounts of citrullinated histone H3. Although we are unable to provide a clear explanation for this finding, we may hypothesize that, at this early time point, 1) DNA release is not a result of NETosis and 2) PAD4-independent mechanisms may be responsible for NETosis. Further studies are necessary to clarify this point.

In this model, roflumilast effects on neutrophil functions correlated with a more rapid weight recovery, which reached the statistical significance at 5 days of infection (Figure 8A). The hypothesis that roflumilast improves the animal well-being by modulating neutrophil recruitment and function was supported by the observation of a direct correlation between the neutrophil number and free DNA with body weight (Supplementary Figure S3). On the contrary, the bacterial load, which was not appreciably modified by roflumilast, was not an influent.

Consistent with this scenario, free DNA accumulated in the BALF of *Pseudomonas aeruginosa*–infected mice positively correlated with neutrophil counts. In this condition, free DNA may be largely represented by NETs, as indicated by histone H3 data. Thus, NET release appears to represent a neutrophil function mainly involved in the pathogenesis of lung inflammation during *Pseudomonas aeruginosa* infection. This interpretation is consistent with recent evidence that free DNA, abundant in CF sputum, shows NET characteristics (Dwyer et al., 2014). Moreover, in the airways of people with CF, NET components, such as elastase and other granule proteins, perpetuate lung damage and inflammation (Marcos et al., 2015; Dittrich et al., 2018), while decondensed chromatin, the main structure of NETs, increases the viscosity of endobronchial secretions, further hampering mucociliary clearance. Clinical observations also confirm the pathogenetic role of NETs in CF, by revealing a positive correlation between the impairment of respiratory function and the level of free DNA or elastase in CF airways, or the level of MPO and antibodies to PAD4 in circulating blood (Marcos et al., 2015; Dittrich et al., 2018; Yadav et al., 2019).

In summary, our study describes a series of pharmacological activities associated with PDE4 blockade in neutrophils, which could be beneficial in a CF clinical setting. Our data, showing that PDE4 inhibitors may sustain a local signal driving neutrophilic inflammation toward physiological resolution, indicate that PDE4 may be a potential novel target to promote a “correction” of neutrophilic inflammation, rather than a complete suppression, which could be detrimental. As PDE4 inhibitors have been recently approved for clinical use in COPD (Rabe, 2011; Wedzicha et al., 2016) and psoriasis (Rich et al., 2016), our present results encourage further research to validate the use of these drugs in patients with CF.

## Data Availability

The original contributions presented in the study are included in the article/Supplementary Material. Further inquiries can be directed to the corresponding authors.
