# Supplementary figures and images for: Type-4 Phosphodiesterase (PDE4) Blockade Reduces NETosis in Cystic Fibrosis
Source: Front Pharmacol. 2021 Sep 8;12:702677. doi: 10.3389/fphar.2021.702677 (PMC8456009; doi:10.3389/fphar.2021.702677)

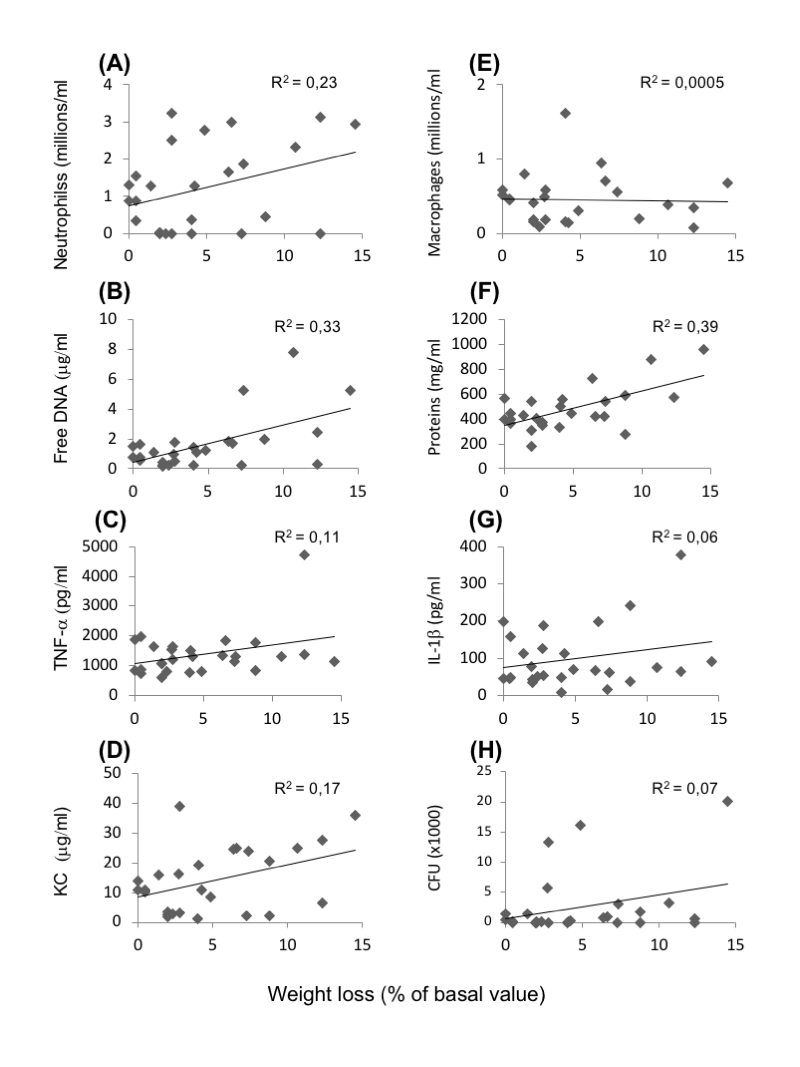

Supplement: Supplementary file 1 [file Image3.TIFF]

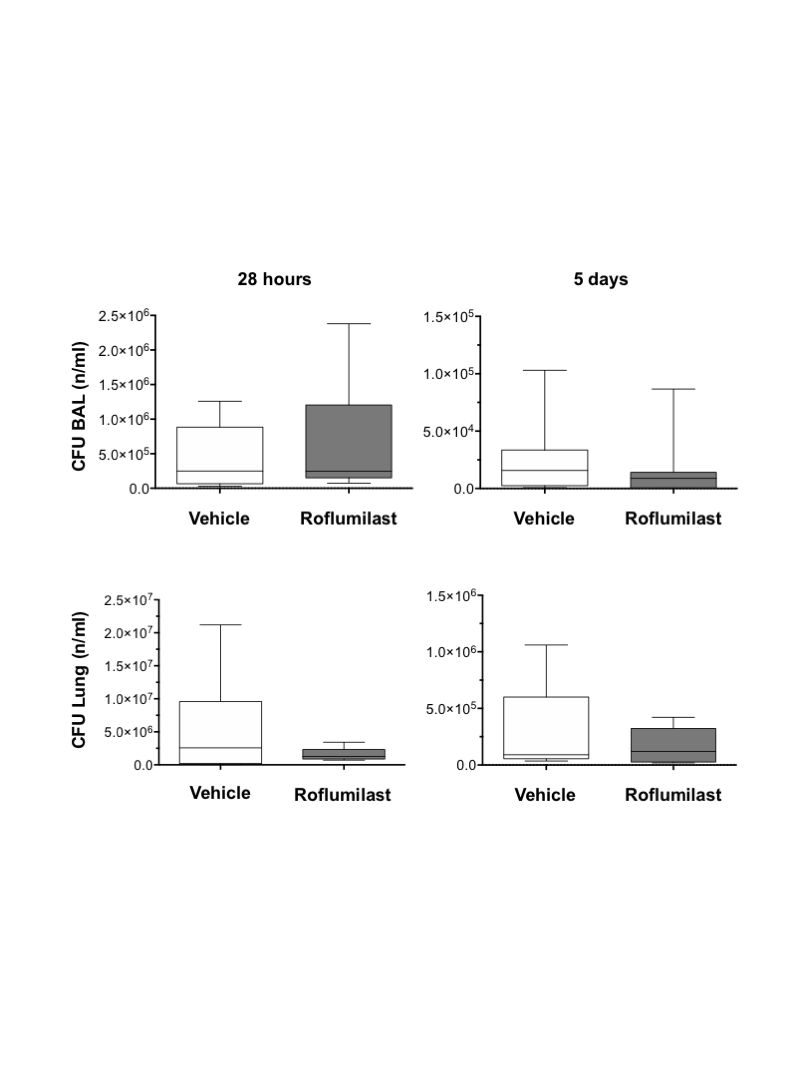

Supplement: Supplementary file 2 [file Image1.TIFF]

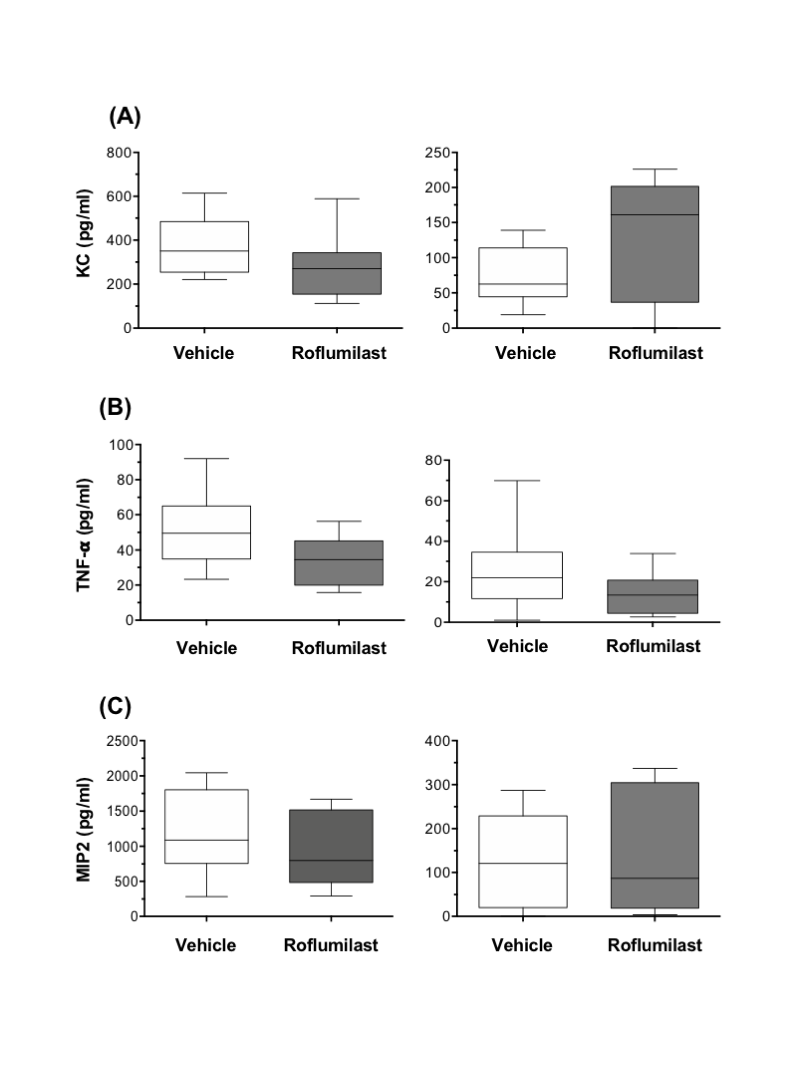

Supplement: Supplementary file 3 [file Image2.TIFF]
