# Supplementary material for: Type-4 Phosphodiesterase (PDE4) Blockade Reduces NETosis in Cystic Fibrosis
Source: Front Pharmacol. 2021 Sep 8;12:702677. doi: 10.3389/fphar.2021.702677 (PMC8456009; doi:10.3389/fphar.2021.702677)
Supplement: Supplementary file 4 [file DataSheet1.docx]

**Supplemental Table 1.** Characteristics of healthy neutrophils remained intact after 18 hours.

| Phenotype | Vehicle | | LPS | | CFTRinh-172 | | LPS + CFTRinh-172 | |
| --- | --- | --- | --- | --- | --- | --- | --- | --- |
| AnnV^-^/P.I.^-^ | 7.2 ± 1.4 | 12.5 ± 4.8 | 14.7 ± 3.8 | 18.2 ± 4.5 | 6.1 ± 2.1 | 11.2 ± 2.9 | 10.2 ± 3.1 | 21.5 ± 5.3 |
| AnnV^+^/P.I.^-^ | 74.0 ± 3.9 | 73.2 ± 4.9 | 60.0 ± 7.1 | 53.2 ± 6.7 | 77.6 ± 3.9 | 67.4 ± 6.2 | 70.3 ± 5.8 | 49.3 ± 10.0 |
| AnnV^-^/P.I.^+^ | 1.5 ± 0.3 | 2.6 ± 0.6 | 0.8 ± 0.2 | 1.8 ± 0.9 | 0.9 ± 0.2 | 2.4 ± 0.8 | 0.7 ± 0.1 | 2.2 ± 0.5 |
| AnnV^+^/P.I.^+^ | 12.8 ± 3.0 | 11.0 ± 2.1 | 14.5 ± 2.7 | 14.3 ± 4.1 | 10.0 ± 1.8 | 11.6 ± 3.1 | 12.8 ± 3.6 | 13.2 ± 3.7 |
|  | DMSO | RNO | DMSO | RNO | DMSO | RNO | DMSO | RNO |

Neutrophils isolated from healthy subjects (n = 8) were treated with RNO (1 mMoles/L) or vehicle (DMSO) and allowed to adhere on fibrinogen-coated surfaces in the presence or absence of CFTRinh-172 (10 mMoles/L), for 18 hours with or without endotoxin. At the end of the incubation, cells were detached by brief exposure to a solution containing 5 mMoles/L EGTA, resuspended in annexin (Ann)V binding buffer, stained with FITC-conjugated-annexinV and propidium iodide (P.I.), as indicated by the manufacturer (ThermoFisher Sientific), and analyzed by Flow Citometry. Intact neutrophils were identified by typical SSC and FSC. Data are mean ± SEM of the relative percentage of cells with the indicated phenotype.

**Supplemental Table 2.** Characteristics of CF neutrophils remained intact after 18 hours.

| Phenotype | Vehicle | | LPS | |
| --- | --- | --- | --- | --- |
| AnnV^-^/P.I.^-^ | 4.2 ± 0.5 | 5.7 ± 6.1 | 16.2 ± 6.1 | 18.5 ± 6.0 |
| AnnV^+^/P.I.^-^ | 82.8 ± 1.1 | 79.4 ± 1.0 | 71.6 ± 5.7 | 68.2 ± 5.8 |
| AnnV^-^/P.I.^+^ | 1.7 ± 0.7 | 2.7 ± 0.7 | 1.8 ± 0.7 | 1.0 ± 0.6 |
| AnnV^+^/P.I.^+^ | 11.3 ± 1.2 | 12.7 ± 0.3 | 10.3 ± 2.1 | 11.3 ± 1.2 |
|  | DMSO | RNO | DMSO | RNO |

Neutrophils from 3 subjects with CF were incubated as described in Supplemental Table 2. Flow cytometric analysis was performed after staining with annexinV (AnnV)/P.I. Data are mean ± SEM of the relative percentage of cells with the indicated phenotype.
